# *De novo* transcriptome assembly for the lobster *Homarus americanus* and characterization of differential gene expression across nervous system tissues

**DOI:** 10.1186/s12864-016-2373-3

**Published:** 2016-01-16

**Authors:** Lara Lewis McGrath, Steven V. Vollmer, Stefan T. Kaluziak, Joseph Ayers

**Affiliations:** Northeastern University Marine Science Center, 430 Nahant Rd, Nahant, MA 01908 USA; Current address: AstraZeneca, 35 Gatehouse Dr, Waltham, MA 02451 USA

**Keywords:** Transcriptome, RNA-Seq, Gene expression, CNS, Nervous system, Central pattern generation, Locomotion, Neuronal differentiation, Lobster, *Homarus americanus*

## Abstract

**Background:**

The American lobster, *Homarus americanus*, is an important species as an economically valuable fishery, a key member in marine ecosystems, and a well-studied model for central pattern generation, the neural networks that control rhythmic motor patterns. Despite multi-faceted scientific interest in this species, currently our genetic resources for the lobster are limited. In this study, we *de novo* assemble a transcriptome for *Homarus americanus* using central nervous system (CNS), muscle, and hybrid neurosecretory tissues and compare gene expression across these tissue types. In particular, we focus our analysis on genes relevant to central pattern generation and the identity of the neurons in a neural network, which is defined by combinations of genes distinguishing the neuronal behavior and phenotype, including ion channels, neurotransmitters, neuromodulators, receptors, transcription factors, and other gene products.

**Results:**

Using samples from the central nervous system (brain, abdominal ganglia), abdominal muscle, and heart (cardiac ganglia, pericardial organs, muscle), we used RNA-Seq to characterize gene expression patterns across tissues types. We also compared control tissues with those challenged with the neuropeptide proctolin *in vivo*. Our transcriptome generated 34,813 transcripts with known protein annotations. Of these, 5,000-10,000 of annotated transcripts were significantly differentially expressed (DE) across tissue types. We found 421 transcripts for ion channels and identified receptors and/or proteins for over 20 different neurotransmitters and neuromodulators. Results indicated tissue-specific expression of select neuromodulator (allostatin, myomodulin, octopamine, nitric oxide) and neurotransmitter (glutamate, acetylcholine) pathways. We also identify differential expression of ion channel families, including kainite family glutamate receptors, inward-rectifying K^+^ (IRK) channels, and transient receptor potential (TRP) A family channels, across central pattern generating tissues.

**Conclusions:**

Our transcriptome-wide profiles of the rhythmic pattern generating abdominal and cardiac nervous systems in *Homarus americanus* reveal candidates for neuronal features that drive the production of motor output in these systems.

**Electronic supplementary material:**

The online version of this article (doi:10.1186/s12864-016-2373-3) contains supplementary material, which is available to authorized users.

## Background

An overarching goal in the study of neurobiology is to translate the capacity to perform an action to its underlying cellular and molecular mechanisms. As a result, nervous systems involved in central pattern generation have become a well-studied model for the control of behavior [1, 2]. Central pattern generators are neural networks that produce repetitive actions such as the beating of the heart or gait patterns in walking or running. They are defined by their ability to produce rhythmic motor patterns even in the absence of sensory feedback [3]. The continuous, measurable physical output of these networks allows for straightforward comparisons between actual behavior and network activity. Moreover, these networks perform complex motor tasks in a coordinated, rhythmic fashion while still adapting the behavior to environmental contingencies [3, 4]. The accessibility of these networks combined with this juxtaposition between precision and flexibility has sparked decades of research on the subject, especially in invertebrate models like the American lobster *Homarus americanus* where these networks are relatively simple (tens or hundreds, rather than thousands, of neurons) compared to their vertebrate counterparts.

In this study, we utilize the American lobster *Homarus americanus* for its role as a model organism in the study of neural networks, though it is also an economically valuable fishery and an important species in marine ecosystem dynamics. To perform this work, we *de novo* assemble a transcriptome for this species, which has no published genome or transcriptome to date. This genetic resource will expand our ability to study this species in both a physiological and ecological context, as well as within the framework of neurobiology and central pattern generation.

**Fig. 1:**
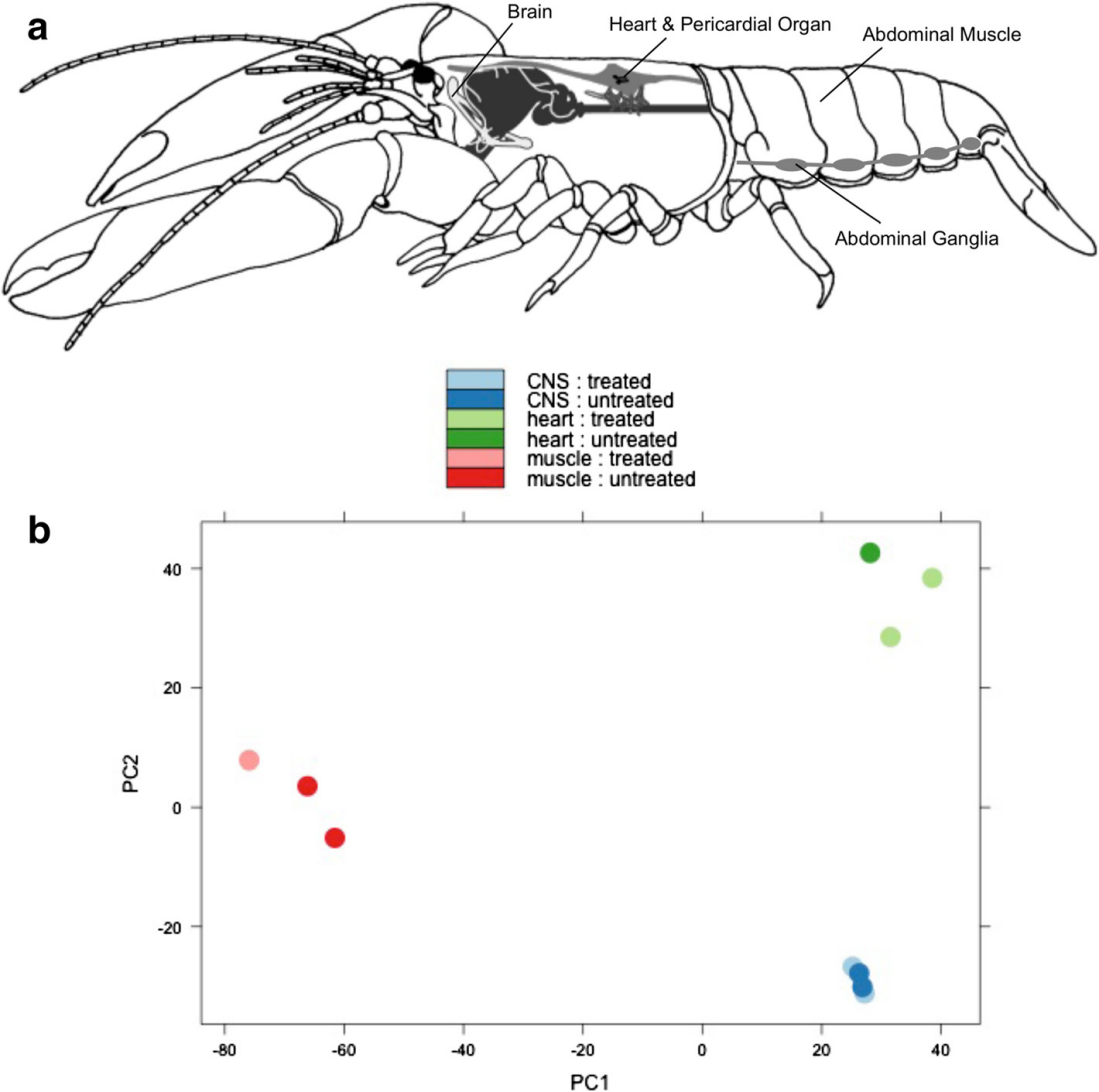
**a** Schematic drawing of the lobster *Homarus americanus*, indicating tissue types collected for this study. Central nervous system tissue samples include either abdominal ganglia (abdominal nerve cord) or the supraesophogeal ganglion (brain). All heart tissue samples include the heart muscle with attached cardiac ganglion and neurosecretory pericardial organ. Muscle tissue samples are a section of the abdominal muscle. Adapted with permission from John Wiley and Sons, from Skiebe 1999. **b** Principal component analysis of transcripts across three tissue types (heart, muscle, and central nervous system tissue) and two treatment levels (hormone treated and untreated). This 2D representation of the first two principal components of these 10 samples demonstrates a greater effect of tissue type than treatment, and also a greater effect of tissue type than variation across individuals. Component 1 explains 87.84 % of the variance (SD = 2.96); component 2 explains 4.08 % of the variance (SD = 0.64)

In the lobster, perhaps the simplest central pattern generating network (CPG) is the cardiac system that controls the rhythmic pumping of the neurogenic heart and distributes hemolymph (or ‘blood’) throughout the body (Fig. 1a). This system includes the continuously bursting cardiac ganglion (neural control center) and motor neuron connections embedded within the heart itself [5]. The cardiac system generates the heartbeat in the frequency range of 0.5-1.5 Hz [6]. Cardiac performance can be influenced by external factors (including temperature and temperature acclimation) [6–8], and by release of neuromodulators from the pericardial organ [9]. The pericardial organ is an important member of both the cardiac and endocrine systems [10]; it is a neurosecretory structure attached to the lateral walls of the pericardial cavity that releases neuromodulators through nerve trunks with dense assemblies of release terminals [5, 11]. These nerves release neuromodulators as hormones into the hemolymph for distribution to other networks and also innervate the heart to directly modulate the cardiac ganglion [12, 13].

Another well-studied model for central pattern generation in the lobster is the abdominal ganglion, which contains several CPG networks that coordinate locomotion and rhythmic escape swimming behaviors and also play a role in posture [14–16]. This system is composed of six abdominal ganglia, one for each abdominal segment, located in the ventral nerve cord. These ganglia control the swimmeret muscles that operate the fin-like swimmerets (located on the ventral side of abdomen) used in locomotion, righting, and ventilation, the slow extensor-flexor muscles used during backward walking, and the large flexor–extensor muscles used for rhythmic escape swimming [14–23]. The abdominal system controls both phasic and tonic muscle fibers and thus, by comparison, can operate in time domains slower and more rapid than the cardiac system [24, 25]. It can maintain ‘background activity’ in maintenance of posture and also generate rapid tail flips or swimmeret beating at frequencies up to 5 Hz [24, 26].

Characterizing the function and connectivity of these circuits led to a control architecture for innate behavior: the command neuron, coordinating neuron, central pattern generator model. These CPG networks are characterized by a particular ability to respond to external and internal variables (temperature, pH) while maintaining the stable performance [1, 2, 7, 27, 28]. It has also led to breakthroughs in our overall understanding of the role of neuromodulation in shaping the activity of neural circuits [29, 30]. Neuromodulators are signaling molecules that act in concert to modify the intrinsic firing properties of neurons, and can transform the functional connectivity of neural circuits and alter their output. Neural activity can be induced, modified, or terminated by input from multiple neuromodulators [30, 31]. Through extensive work, researchers have identified dozens of neuromodulators in crustacean decapods and characterized their ability to alter circuit dynamics in vitro and, in some cases, motor activity or behavior in vivo [29]. Neuromodulators can be released both intrinsically (from a cell within the circuit) and extrinsically (from another area of the nervous system, such as the neurosecretory pericardial organ) (Fig. 1a). However, despite evidence of systemic release of neuromodulators, they are not ubiquitous within the nervous system—immunocytological work and, recently, a characterization of the *H. americanus* peptidome across tissue types suggests localized distribution of neuromodulators [32]. Exploring the full specificity of expressed neuromodulators and, particularly, their receptors across nervous system tissues is an important next step towards a more complete understanding of the complex interplay of neuromodulators in functional motor networks.

Accordingly, the activity of a neural network is not just conditional to modulatory inputs, but also to the response of the participating neurons to these inputs. The response is dictated by intrinsic properties of those neurons—the number and kind of ion channels on the membrane [33]. Thus, recent focus has shifted to answering the question: what genetic constructs underlie the production of these stereotyped motor patterns? Current theoretical and molecular research demonstrates correlations between gene expression of different ion channel proteins are actively regulated to maintain robust neuronal output [34–40]. In the invertebrate CPGs the stomatogastric and cardiac ganglia, despite variable expression levels in a particular ion channel, there exist characteristic sets of correlated expression of these genes. The relationships between potassium channels (*shal* I^A^, *shab* I^Kd^, *shaw* I^Kd^, *shaker* I^A^ and *BKKCa* I^K[Ca])^) and membrane conductances in identified cell types are particularly well described [34–36, 39]. Though these features are only a small portion of the range of factors governing the identity of these networks, they do speak to the overall trend in opinion that neuronal identity is not defined by the expression of unique genes, but by specific combinations of genes [41].

In this study, we characterize the transcriptional profiles of two types of nervous system tissues—motor, sensory, and command neuron tissue from the abdominal ganglia and supraesophogeal ganglia (or ‘brain’), and hybrid neural/muscle tissue from the heart. We characterize these central nervous system (CNS) and ‘hybrid’ heart tissues against muscle tissue and against each other to address representative transcriptomic signatures of neural tissue types. Finally, we compare abdominal ganglia to the heart tissues to target transcriptome-wide differences between these two central pattern generating tissues.

These experiments also explore the role of a circulating neuromodulator in the transcriptional regulation of ion channels by including a hormonal treatment with proctolin. Proctolin is an endogenous pentapeptide that acts as an excitatory neuromodulator [42]. It was selected for this study because its physiological role as a neuromodulator is well characterized. Proctolin can increase the frequency of action potentials, increase the amplitude of muscle contraction, and initiate activity in quiescent systems [43–48]. It is also accepted to function at a system-wide hormonal level, a context in which this neuropeptide remains poorly understood [49, 50]. Here, we investigate the role of proctolin as a hormonal regulator of gene expression.

The experiments in this study were performed by sampling three types of tissue: muscle (abdominal muscle, *n = 3*), neural (abdominal nerve cord, *n = 3*; supraesophogeal ganglion, *n = 1*), and neuromuscular hybrid (heart, *n = 3*). Individuals used were treated with three daily exogenous proctolin injections to temporarily raise hemal concentrations of this neuromodulator in vivo to 10^−6^M (treated, *n = 5*) or injection of physiological saline (control/untreated, *n = 5*). The effects of increased hemal proctolin across treated and untreated tissues are limited and addressed briefly. All other analyses are conducted bioinformatically controlling for the effects of this treatment in our differential gene expression analysis, and reflect only changes in tissue type (Additional file 1: Table S1). For analyses of tissue type we include both central pattern generating abdominal ganglia and descending inputs (brain) to characterize the differences between these CNS tissues and muscle or heart tissues. However, for direct comparison of the abdominal and cardiac networks, differential gene expression analysis was re-run with only the abdominal ganglia and heart samples.

By employing a transcriptome-wide approach to investigate neural tissue types and examine two central pattern generating networks, we aim to distinguish combinations of factors involved in determining neuronal identity and function of these systems, including ion channels, neurotransmitters, neuromodulators, receptors, and other gene products.

## Results and discussion

### *De novo* transcriptome assembly and annotation

To date, there remains no published genome for *Homarus americanus*. Thus, for this study, we *de novo* assembled and blast annotated a reference transcriptome for *H. americanus* in Trinity using 119.7 million reads from four tissue types (heart, abdominal muscle, abdominal nerve cord, brain) stemming from four lobsters. The assembly resulted in a transcriptome of 115,757 contigs, with an N50 of 1,289. The maximum and minimum contig lengths were 17,481 and 201 bp, respectively, with approximately 25 % of the contigs exceeding 1,000 bp in length. tBlast against NCBI and UniProtKB’s Swiss-Prot/TrEMBLE databases resulted in reliable protein annotations (e value < 10^−4^) for 34,813 contigs, or approximately 30 % the assembled contigs. We observed the N50 in annotated (1909bp) and unannotated (773bp) transcripts varied in length, suggesting that larger transcripts were more likely to have lower blast e-values and thus better protein annotations.

Of our 34,813 annotated transcripts, the annotations contained 12,389 unique proteins, an approximation determined by redundancies in Entrez Gene IDs. Mapping Entrez Gene IDs to Gene Ontology (GO) annotations identified 11,383 GO categories represented in our transcriptome, including 7,161 gene products attributed to biological processes, 1,090 attributed to cellular components, and 3,132 attributed to molecular function. The GO terms attributed to the greatest number of genes, in descending order, were nucleus (GO:0005634), cytoplasm (GO:0005737), protein binding (GO:0005515), integral component of membrane (GO:0016021), membrane (GO:0016020), metal ion binding (GO:0046872), and plasma membrane (GO:0005886).

### Differences in expression between all tissue types and treatment groups

Samples were visualized using a principal component analysis and sample-to-sample distances as data quality assessment and also to visualize relative relatedness between samples (Fig. 1b). Principal component analysis and sample-to-sample distances (Additional file 2: Figure S1) display a closer relation of transcripts within a given tissue type than within a hormone treatment group. Principal component analysis also closely clusters the two types of neural tissues represented, the supraesophogeal (brain) and abdominal ganglia (hereafter together referred to as CNS nervous system or nerve tissues). In Additional file 2: Figure S1, a simple representation of Euclidian distances demonstrates nervous system tissues are farther from muscle and heart tissues than muscle and heart tissues are from each other.

### Differential expression between treated and untreated tissues

Three daily treatments elevating hemal levels of the neuropeptide proctolin resulted in significant differential expression (adj *p* < 0.05) of 255 transcripts. Of these transcripts, 79 had reliable protein annotations (31 %). Gene ontology (GO) enrichment analysis did not identify significantly overrepresented (SO) GO categories (adjusted *p* value < 0.05, BH method) in treated tissues. Of the annotated transcripts, 80 % were upregulated and largely included proteins involved in immune and neural systems. Neural transcripts included innexin, proteins involved in the gap junctions between electrically connected neurons (adj *p* < 0.05, +3 log_2_fold change), and transient receptor potential channels (TRPA1, TRP pyrexia) (adj *p* < 0.05, +3 log_2_fold change). The greatest log_2_fold change was observed in three transcripts annotating to anti-lipopolysaccharide factors (ALFs) (adj *p* < 0.001, +5 log_2_fold change), a peptide with potent anticoagulation and antimicrobial abilities.

ALFs have been identified in the lobster and other crustaceans as part of the innate immunity of these organisms, a system that largely takes place in the hemocytes where antimicrobial compounds are synthesized and stored for release into the hemolymph [51–53]. These results indicate a relationship between hemal proctolin and increased synthesis of antimicrobial proteins.

Though an immune response could have been evoked by the injections themselves, we believe the immune response is likely not an artifact of treatment given the untreated and treated lobsters were injected in the same method with either sterile physiological saline or an identical solution containing synthetic proctolin, respectively. Moreover, the connection between another neuromodulator, octopamine, and immune responses is well documented in invertebrate systems [54]. Octopamine increases total hemocyte count and nodule formation, enhances inositol trisphosphate (IP_3_) production in hemocytes, and increases phagocytosis [55–57]. The neuromodulator serotonin (5-Hydroxytryptamine) is also known to regulate phagocytosis, hemocyte production, and nodule formation [56–58]. The ability of octopamine to regulate immune responses is mediated by G-protein coupled receptors (GPCRs) and second messenger systems. The identified receptor for proctolin is also a GPCR, suggesting a similar mechanism by which proctolin may regulate immune response [59]. Here we see hemal proctolin levels upregulate the transcription of ALFs, as well as a stress activated protein kinase identified in another crustacean immune response [UniprotKB: G0ZJ53] (adj *p* < 0.001, +4 log_2_fold change) [60]. These results implicate a novel role for hemal proctolin in innate immunity pathways, and suggest this neuromodulator may be multi-faceted in its role as a signaling molecule and act on both neural and immune systems.

### Differential expression between heart and muscle tissues

We found 15,046 transcripts with significant differential expression (adj *p* < 0.05) in heart tissues when compared to abdominal muscle tissues; of these differentially expressed (DE) transcripts, 32 % had reliable protein annotations (Table 1). GO enrichment analysis indicated 11 SO categories in heart tissues. The SO categories included extracellular region (GO:0005576), myoblast fusion (GO:0007520), actin binding (GO:0003779), structural constituent of muscle (GO:0008307), calcium ion binding (GO:0005509), and response to heat (GO:0009408).

**Table 1:** Number of transcripts out of 115,757 total transcripts with significant differential expression (adj *p* <0.05) across tissue types

Contrast	Differentially expressed transcripts	Annotated transcripts	Unique annotations
Heart vs. Muscle	15,046	4,839	2,557
Nerve vs. Muscle	28,479	10,033	4,755
Nerve vs. Heart	20,179	7,388	3,797

Annotated transcripts depicts the number of differential expressed (DE) transcripts with a reliable protein annotation. Unique annotations indicate the number of unique genes within the annotated DE transcripts, as determined by associated Entrez Gene ID numbers

### Differential expression between central nervous system and muscle tissues

Results indicate 28,479 DE transcripts (adj *p* < 0.05) in CNS tissues (abdominal and supraesophogeal ganglia) compared to muscle tissues; 35 % annotated and included 4,755 unique genes. These genes comprised 22 significantly overrepresented GO categories (adj *p* <0.05), including plasma membrane (GO:0005886), cell adhesion (GO:007155), extracellular region (GO:005576), axon guidance (GO:007411), synapse (GO:0045202), and neurotransmitter secretion (GO: 0007269).

### Differential expression between central nervous system and heart tissues

We found 20,179 DE transcripts between nerve and heart tissues, including 7,388 with protein annotations (37 % of all DE transcripts), 3,797 unique genes, and 38 significantly overrepresented GO categories (*p* < 0.05). Enriched GO categories included plasma membrane (GO:0005886), structural constituent of ribosome (GO:0003735), axon guidance (GO:0007411), extracellular matrix (GO: 0031012), and translation (GO: 0006412).

**Fig. 2:**
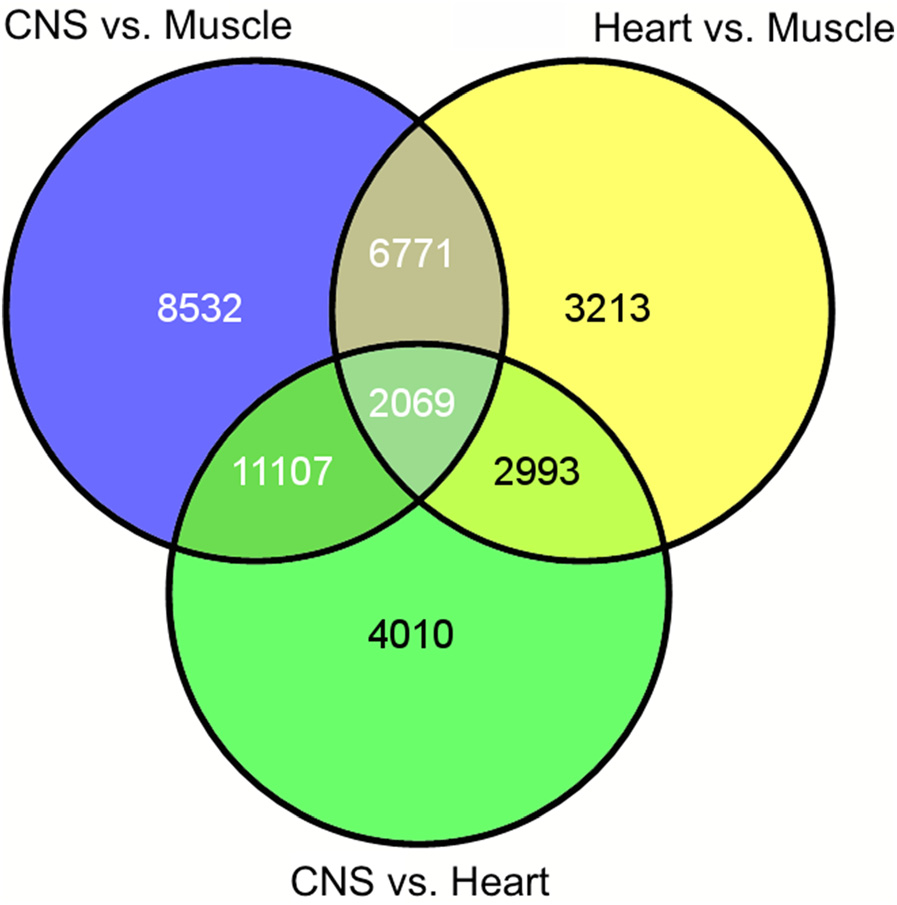
Venn diagram of differentially expressed transcripts by tissue comparison. Values indicate number of transcripts with significant differential expression (*p* value < 0.05) across that contrast (heart vs. muscle tissues; central nervous system tissues vs. muscle tissues; central nervous system tissues vs. heart tissues)

To address which genes were consistently DE in the neural and heart tissues compared to muscle tissues, we constructed a venn diagram (Fig. 2). Overlap of DE transcripts identified 6,771 transcripts differentially expressed across both contrasts and 2,069 DE across all three contrasts; these groups included many of the nervous system genes described in more detail below.

### Regulation of neurotransmitters and neuromodulators

**Fig. 3:**
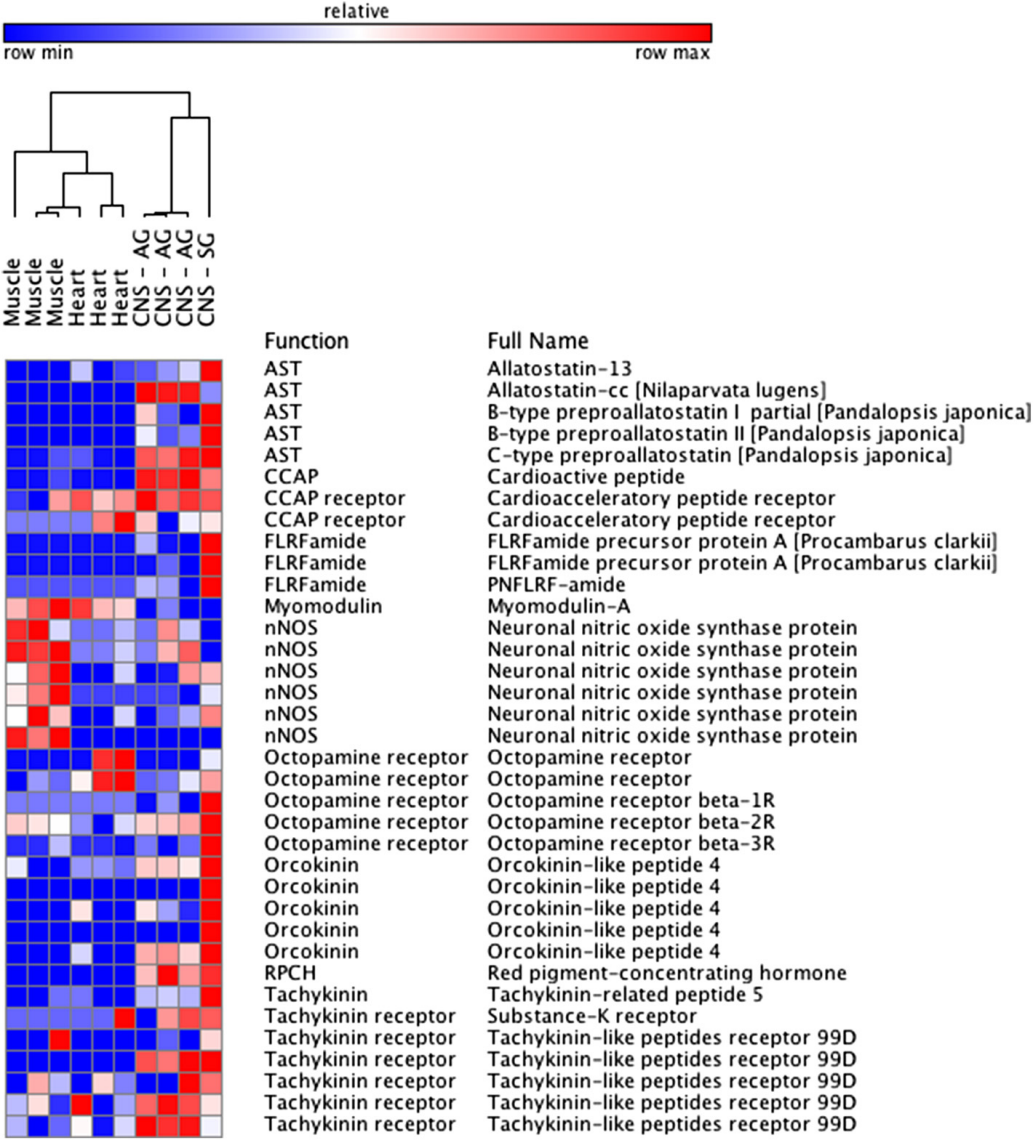
Heatmap of select neuromodulators and receptors with tissue specific expression patterns. Relative to each other, red cells indicate high levels of expression and blue cells indicate levels of low expression. For central nervous system tissues, AG – abdominal ganglion, SG – supraesophogeal ganglion

The nervous system of crustaceans is rich in neuromodulatory substances that act as extrinsic and intrinsic regulators of nervous system activity. To date, over two dozen of these neuroactive molecules have been identified [61]. Our goal was to assess the presence and relative expression of these known crustacean neurotransmitters and neuromodulators across neural and non-neural tissues. Proteins involved in signaling pathways were detected for 20 different neuroactive substances, including known crustacean neurotransmitters (acetycholine, glutamate, serotonin, dopamine) and neuromodulators allostatin (AST), FLRFamide, FMRFamide, cardioactive peptide (CCAP), octopamine, orcokinin, dopamine, etc. These proteins included neuropeptides, receptors, and enzymes involved in neurotransmitter pathways (i.e. synthesis). As expected, most of these genes were upregulated in the nervous system tissues compared to muscle tissues (Table 2). However, some neuromodulators exhibited tissue-specific expression signatures for particular neuromodulator sequences or receptors (Fig. 3). For instance, the neuropeptide myomodulin was DE and downregulated in nervous tissue compared to both muscle (*p* < 0.05, −7 log_2_fold change) and heart (*p* <0.05, −6 log_2_fold change) tissue, suggesting a localized role in neuromuscular signaling. There was also a bifurcation of regulation of neuronal nitric oxide synthase (NO) across nerve and muscle tissues, suggesting the proteins or protein isoforms involved in NO signaling differ across tissues. NO is of particular interest as it has recently been shown to gate the polarity of endocannabinoid-modulation to shift the excitation-inhibition balance of a synapse and thus mediate long-term potentiation of rhythmic locomotor circuits [62–65]. All six transcripts for the carboxyl-terminal PDZ ligand of neuronal nitric oxide synthase protein (70729) were downregulated in nerve tissue compared to muscle, and three of the six were DE (*p* < 0.05). Three nostrin (115677, 521834) transcripts were DE and downregulated (−5 log_2_fold change or greater) across the tissue types, but five other nostrin transcripts were DE and upregulated (+2 log_2_fold change or greater).

**Table 2:** Expression of known crustacean neuromodulators and neurotransmitters in the *Homarus americanus* transcriptome

Neuromodulator	Function	Count	DE	Up	Down
5-HT	5-HT receptor	17	4	4	0
5-HT	5-HT transporter	3	1	1	0
Acetylcholine	ACH receptor	35	13	13	0
Acetylcholine	ACH transporter	3	2	2	0
Angiotensin	Angiotensin	16	7	1	6
AST	AST	5	4	4	0
AST	AST receptor	2	0	0	0
Callisulfakinin	Callisulfakinin	1	0	0	0
CCAP	CCAP	1	1	1	0
CCAP	CCAP receptor	2	1	1	0
Dopamine	Conversion of dopamine to noradrenaline	2	2	2	0
Dopamine	Dopamine receptor	6	3	3	0
Dopamine	Dopamine transporter	2	0	0	0
FLRFamide	FLRFamide	3	1	1	0
FMRFamide	FMRFamide receptor	7	1	1	0
FMRFamide	FMRFamide-related neuropeptide	5	2	2	0
GABA	GABA receptor	22	4	0	4
GABA	GABA transporter	12	10	7	3
Glutamate	Excitatory amino acid transporter	11	5	5	0
Glutamate	Glutamate receptor	126	52	37	15
Glutamate	Glutamate transporter	11	6	6	0
HCNP	HCNP	1	1	1	0
Myomodulin	Myomodulin	1	1	0	1
NO	Nitric-oxide (NO) synthesis regulation	29	11	5	6
Octopamine	Octopamine receptor	5	0	0	0
Orcokinin	Orcokinin	5	2	2	0
Prohormone-1	Brain peptide SYWKQCAFNAVSCF-amide	1	1	1	0
RPCH	RPCH	1	1	1	0
SIFamide	SIFamide receptor	1	0	0	0
Substance K	Substance-K receptor	1	0	0	0
Tachykinin	Tachykinin	1	1	1	0
Tachykinin	Tachykinin receptor	5	3	3	0

Transcripts manually curated into functional categories by their annotations. Count is the number of transcripts mapping to a function; DE indicates the number of transcripts differentially expressed in nervous system tissues compared to muscle tissues (adj *p* < 0.05); up and down signify the number of DE transcripts upregulated (positive log_2_fold change) or downregulated (negative log_2_fold change), respectively

In comparing the heart tissue (which contains both muscle and the cardiac ganglion nervous system) with the other nervous tissues (abdominal chain and brain), there was a contrast in the expression of different octopamine receptors. Octopamine receptors beta 1-R, 2-R, and 3-R were upregulated in other nervous system tissues, compared to heart tissues (*p* value, *ns,* +2 log_2_fold change or greater). In contrast, both octopamine receptors transcripts matching UniProtKB Q25188.1 of *Heliothis virescens* were downregulated (1 of 2 transcripts DE, *p* value < 0.05, −2 log_2_fold change) and thus more highly expressed in cardiac tissue (Fig. 3). Octopamine *β-*receptors are a group of receptors homologous with vertebrate *β*-adrenergic receptors [66]. These receptors respond to octopamine by increasing intracellular cAMP but display disparate pharmacological profiles, suggesting differences in sequences between these receptors results in varying functional roles in signaling activities [66, 67]. Our data provide context for localization of expression and expression levels in *H. americanus*, and suggest differences in receptor sequences for octopamine (as well as other signaling molecules) may play a role in defining specificity in targets and effects of these molecules as a circulating neuromodulators.

### Transcription Factors for Neuronal Differentiation

To identify candidates for transcription factors involved in the differentiation of our neuronal tissues, we performed a blast analysis for transcription factors identified in terminal selector programs, or pathways that that control the expression of identifying features of mature individual neuron types [33, 68–72]. We searched for 24 different gene sequences, all of which were found within our transcriptome (e-val < 10^−4^) (Additional file 1: Table S2). Of these sequences, we found several transcription factors with a high proportion (>40 %) of transcripts DE and upregulated in our nervous system tissues, including *ceh-36* the Otx-type homeobox gene involved in chemosensory neuron differentiation, *ttx-1* an Otx-type transcription factor involved in thermosensory neuron identity, and *ets-5* an ETS domain transcription factor involved in CO_2_/O_2_ sensory neurons [71].

We also blasted our transcriptome for the homeodomain transcription factor Shox2, which has been linked to excitatory interneurons in the rhythmic pattern generating kernel for spinal locomotion in mice [73]. The top hit (comp32264_c0_seq2) had a 99 % sequence identity match to Shox2 and was DE and upregulated in heart tissues compared to muscle tissue (adj *p* < 0.05, +6 log fold change) and downregulated in nerve tissues compared to heart tissues (adj *p* < 0.05, −7 log fold change), indicating this transcription factor is highly expressed in our cardiac network compared to the abdominal ganglia, brain, or muscle.

### Candidate genes for differentiation between abdominal and cardiac CPGs

To identify divergent genetic signatures across the abdominal and cardiac central pattern generating networks, we conducted a different gene expression analysis of just abdominal nerve cord tissues (*n* = 3) over heart tissues (*n* = 3) and then examined transcripts involved in neuronal identity.

**Fig. 4:**
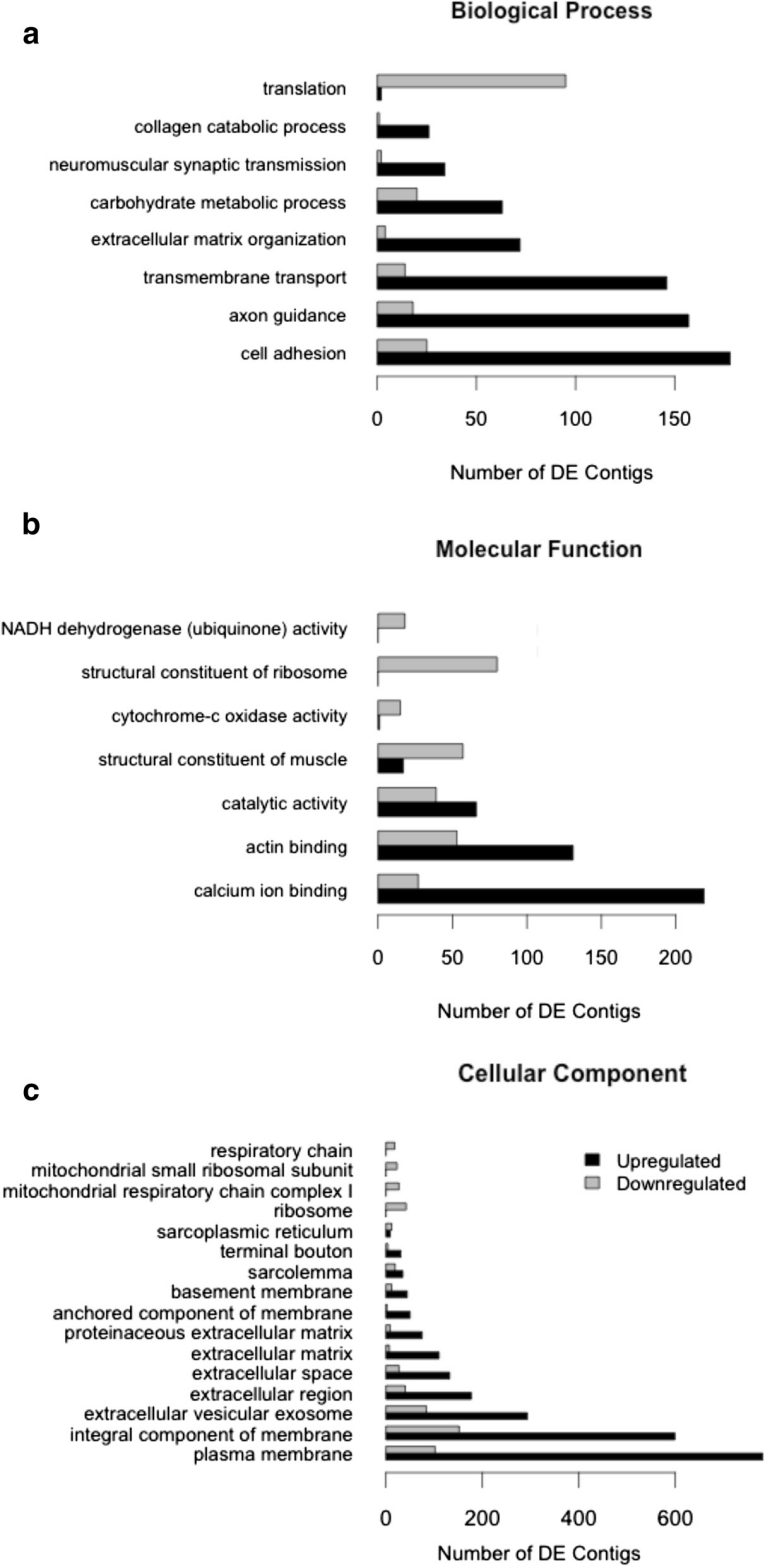
Gene ontology analysis of abdominal nerve cord vs. heart tissue. Analysis is divided by category: **a**. Cellular Component. **b**. Biological Process. **c**. Molecular Function. Each panel includes only significantly enriched GO terms (adjusted *p* value < 0.05) and depicts number of differentially expressed contigs (*p* < 0.05) that are upregulated (black bars, positive log2foldchange) or downregulated (gray bars, negative log2foldchange). For full list of GO terms with ID numbers, refer to Additional file 1: Table S3

In summary, 14,955 transcripts were differentially expressed; 38 % of transcripts had protein annotations. Gene ontology analysis identified 31 significantly enriched GO categories (*p* < 0.05) (Fig. 4). Many of these results, like the structural constituent of muscle (GO:0008307) and respiratory chain (GO:0070469), were downregulated in the abdominal nerve cord and are attributable to differences in nervous system tissue versus mixed muscle/nervous system tissue (Additional file 1: Table S3). Thus our analysis focused on GO categories and genes involved in neuronal differentiation with the goal of addressing the differences in the CPG nervous systems of the cardiac ganglion and abdominal ganglia.

**Fig. 5:**
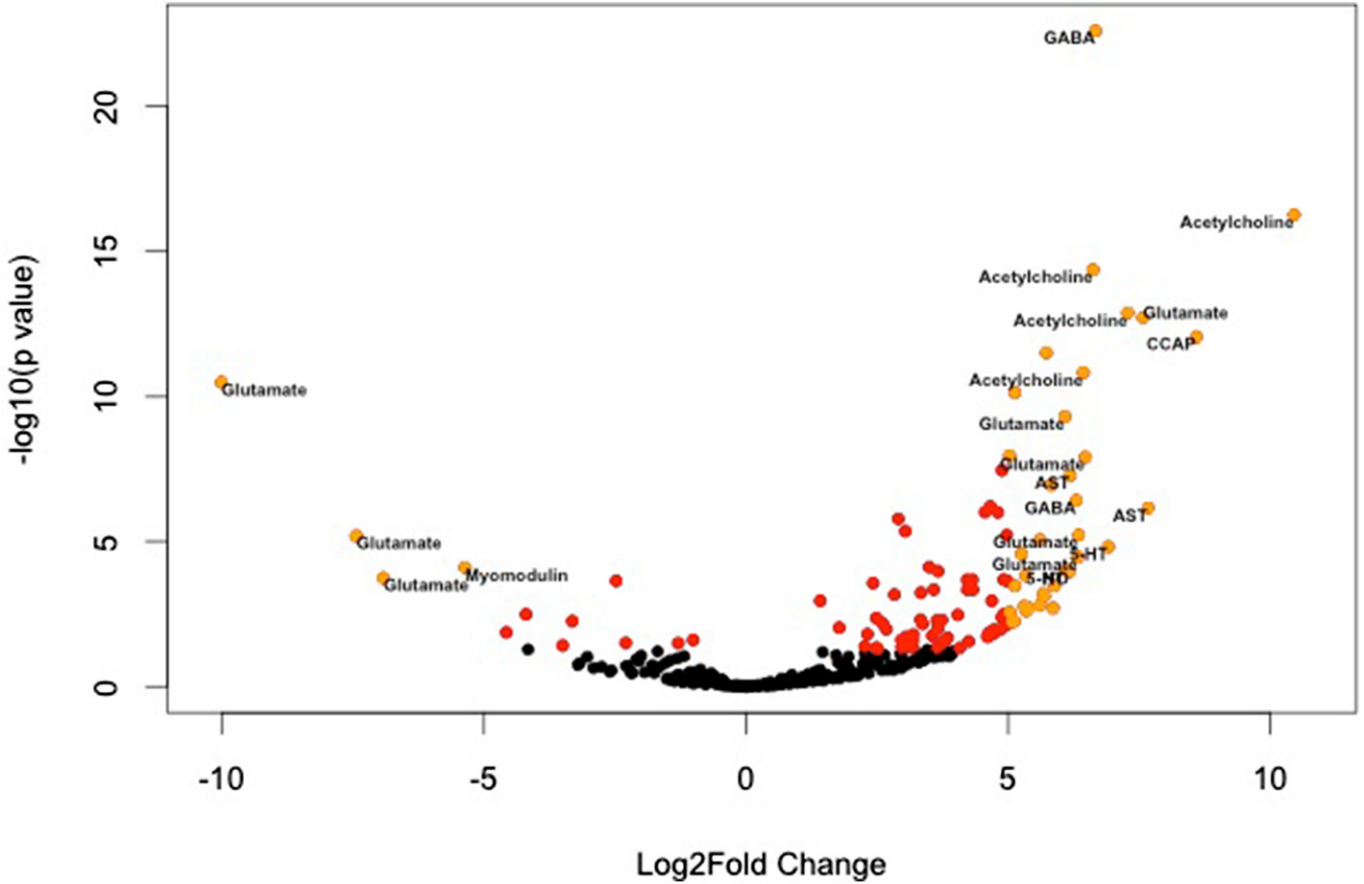
Analysis of neurotransmitters, neuromodulators, and their receptors in the abdominal nervous system compared to the heart system. Red dots indicate significantly DE contigs (p < 0.05). Orange dots indicate DE contigs with log_2_fold change greater than 5 (upregulated in abdominal system) or less than −5 (downregulated in abdominal system compared to heart)

**Fig. 6:**
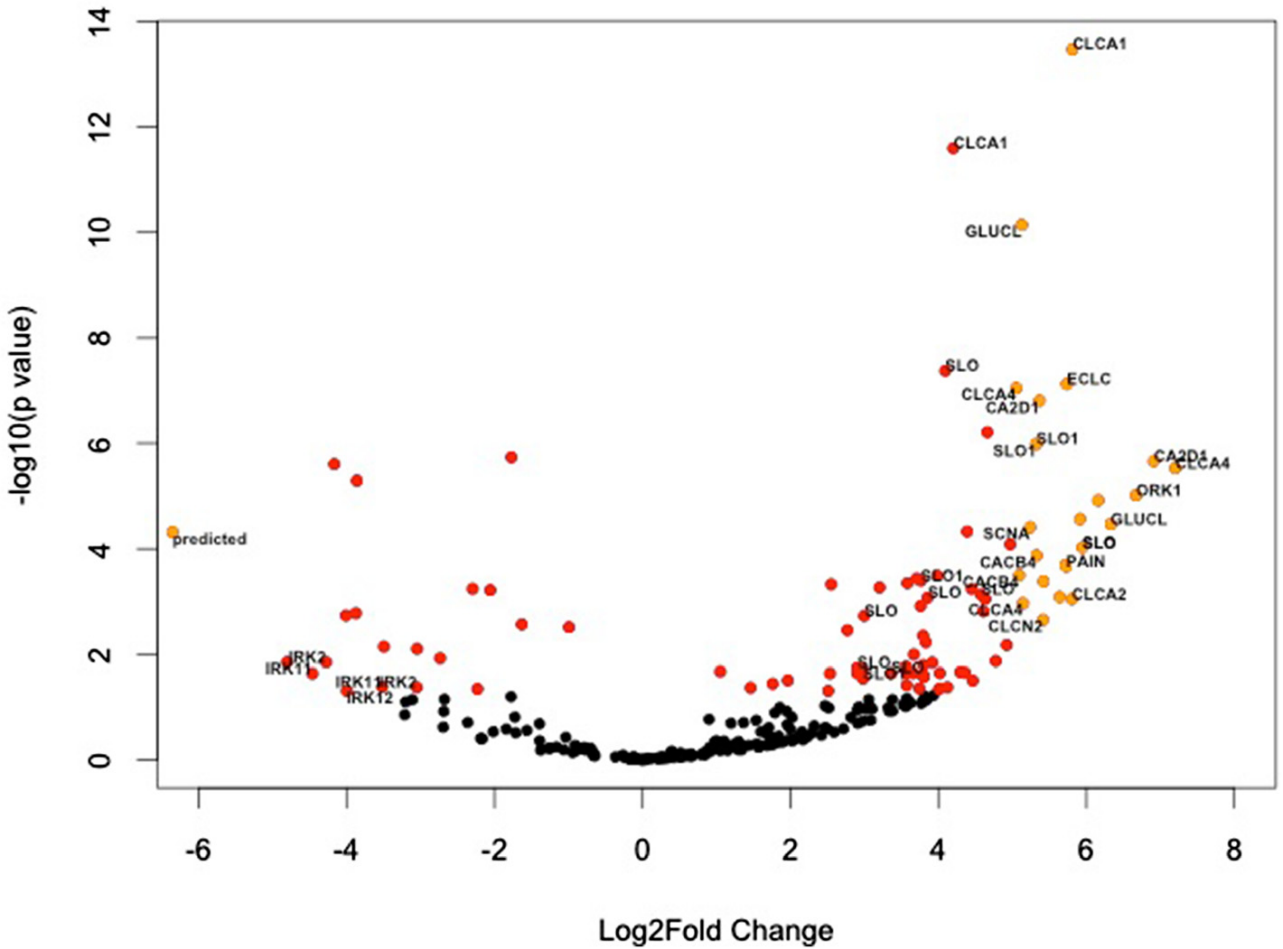
Analysis of membrane channels in the abdominal nervous system compared to the heart system. Red dots indicate significantly DE contigs (p < 0.05) annotating to known membrane channels. Orange dots indicate DE contigs with log_2_fold change greater than 5 (upregulated in abdominal system) or less than −5 (downregulated in abdominal system compared to heart)

Enriched GO category terminal bouton (GO:0043195) identified a role in kainite family glutamate receptors (GluK) in differentiation of these CPGs. GluK 2 receptors are DE and upregulated in abdominal ganglia (GluK2 p <0.001, +6 log_2_fold change) whereas GluK 3 and GluK 5 are DE and downregulated (respectively, GluK3 *p* <0.001, −10 log_2_fold change and GluK5 *p* < 0.05, −5 log_2_fold change). We also employed previous techniques for identifying neuromodulators, ion channels, and receptors (Figs. 5 and 6). Overall, this analysis detected 421 transcripts annotating to membrane ion channels or receptors. Results indicated cardioactive peptide (CCAP) is upregulated (*p* < 0.001, +9 log_2_fold change) in the abdominal system compared to the heart. This result is particularly interesting given the nerve terminals in the heart tissues from the neurosecretory pericardial organ; the expression of CCAP was first discovered in the pericardial organs, hence the etymology [74]. Here, CCAP is indeed expressed in the heart tissues, but has relatively greater expression in the abdominal system. Also upregulated in the abdominal system were two variants of allostatin annotating to allatostatin-cc (*Nilaparvata lugens*) (*p* < 0.001, +7 log_2_fold change) and C-type preproallatostatin (*Pandalopsis japonica*) (*p* < 0.001, +6 log_2_fold change) and the neuromodulator red pigment containing hormone (RPCH) (*p* < 0.05, +4 log_2_fold change) (Fig. 5). Several contigs of acetylcholine pathway proteins were also upregulated in the abdominal CPG including subunit alpha 6 (*p* < 0.001, +10 log_2_fold change) and acetylcholinesterase (*p* < 0.001, +7 log_2_fold change). Myomodulin A (*p* < 0.001, −5 log_2_fold change) and octopamine receptors (*p* < 0.05, −2 log_2_fold change) were downregulated in the abdominal system compared to the cardiac system. Collectively, these results indicate CCAP, AST, RPCH, GluK2, and acetylcholine as strong candidates for the function of abdominal networks, and GluK3, GluK 5, myomodulin and octopamine as signatures of the cardiac network.

We observed all voltage gated L-type Ca^2+^ channel subunits and Ca^2+^ channel subunit alpha-2/delta-1 are DE and upregulated in the abdominal ganglia (Table 3). We also observed glutamate-gated Cl^−^ channels (GLUCL) and calcium-activated Cl^−^ channels (CLCA) were largely DE and upregulated, with log_2_fold changes of many transcripts greater than +5 (Fig. 6). K^+^ channels slo-1/slowpoke are also DE and upregulated. These large-conductance, big potassium or ‘BK’ channels are involved in calcium-dependent K+ currents (I_KCa_), participate in repolarization of the presynaptic terminal, and are important to the timing of action potentials [75–77]. Interestingly, recent studies in *Drosophila melanogaster* with *slowpoke* knockout mutants and RNAi have identified timing deficiencies in rhythmic motor patterns and, particularly, a drastically decreased ability for *slo* mutants to initiate rhythmic flight activity [78, 79]. Taken with our observed expression increases in the abdominal system, future studies could explore whether the *slo* K+ channel is a key constituent in intermittently active, rather than continually active, rhythmic motor generating nervous systems.

**Table 3:** Membrane channels differentially expressed across the abdominal and cardiac nervous system tissues.

Type	Abbreviation	Annotation	Count	DE	Up	Down
Ca	CA2D1	Voltage-dependent Ca channel subunit alpha-2/delta-1	3	3	3	0
Ca	CACB2	Voltage-dependent L-type calcium channel subunit beta-2	3	3	3	0
Ca	CACB4	Voltage-dependent L-type calcium channel subunit beta-4	3	3	3	0
Cl	CLCA1	Calcium-activated chloride channel regulator 1	9	5	4	1
Cl	CLCA2	Calcium-activated chloride channel regulator 2	13	8	7	1
Cl	CLCA3	Calcium-activated chloride channel regulator 3	2	1	1	0
Cl	CLCA4	Calcium-activated chloride channel regulator 4	7	3	3	0
Cl	CLCN2	Chloride channel protein 2 ClC-2	5	4	4	0
Cl	ECLC	Epithelial chloride channel protein	6	4	3	1
Cl	GLUCL	Glutamate-gated chloride channel	18	5	5	0
K	KCNAL	Potassium voltage-gated channel protein Shal	1	1	1	0
K	KCNKA	Potassium channel subfamily K member 10	2	1	1	0
K	ORK1	Open rectifier potassium channel protein 1	4	2	2	0
K	SLO	Calcium-activated potassium channel slowpoke	16	7	7	0
K	SLO1	Calcium-activated potassium channel slo-1	3	3	3	0
Na	SCNA	Sodium channel protein para	14	6	6	0
Cl	predicted	Epithelial chloride channel protein-like (Gallus gallus)	1	1	0	1
K	IRK2	Inward rectifier potassium channel 2	2	2	0	2
K	IRK11	Inward rectifier potassium channel 11	2	2	0	2

Count is the number of transcripts annotating to a channel type; DE indicates the number of transcripts differentially expressed in abdominal compared to heart tissues (adj *p* < 0.05); up and down signify the number of DE transcripts upregulated (positive log_2_fold change) or downregulated (negative log_2_fold change), respectively

Ion channels that were DE and downregulated in this comparison, thus displaying significantly increased expression in cardiac systems, identified a predicted Cl^−^ channel and two inward-rectifying K^+^ (IRK). IRK channels are important to neuronal excitability and the timing of neuronal activity [80, 81]. Combined, these observations suggest families of channels critical to the function and timing of motor pattern generating circuits including IRK channels for the heart and *slo* family BK channels and CLCA channels for the abdominal system. For a full list of differentially expressed ion channels, please refer to Additional file 1: Table S4.

### Heart tissue in response to heat

An unexpected result of this study was the identification of significant overrepresentation of thermosensory genes in the heart tissues. Interestingly, in GO enrichment analysis of heart vs. muscle tissues the only significantly overrepresented biological process was ‘response to heat’ (GO:0009408), with over 50 % of involved genes differentially expressed (18 of 32 genes in category, *p* < 0.05). For visualization of enriched biological processes in heart tissues, please refer to Additional file 3: Figure S2. 

As mentioned, central pattern generators are characterized by their ability to maintain robust output across changing internal and environmental factors [27, 82]. For poikilotherms, this ability is particularly important given there are no internal mechanisms for regulating temperature—an environmental factor with undeniable effects on all biological processes. The thermal behavior of *H. americanus* and other decapod crustaceans is well documented. They are able to respond to small changes in temperature (<0.5° C) and maintain rhythmic nervous system function over large temperature ranges [8, 82–84]. Moreover, these organisms are thermotactic and navigate toward preferred temperatures to optimize physiological function [85–87]. Our results may indicate candidate genes for this precise response to temperature in the cardiac and central nervous system tissues (Table 4). Of the 18 DE genes involved in heat response, two were DE and downregulated in all contrasts: heat shock 18 kDa protein and protein Efl21. Additionally, TRP channel pyrexia, locomotion defects protein, and hippocampal cholinergic neurostimulating peptide, were DE and upregulated in both heart and nervous system tissues when compared to muscle tissues.

**Table 4:** The significantly differentially expressed genes in heart tissues in the “response to heat” GO category (GO:0009408) (heart tissue vs. muscle tissue, adj *p* <0.05)

Gene ID	Annotation	Log_2_ fold change
38037	Transient receptor potential channel pyrexia	5.4
3771872	Heat shock protein 67B2	4.4
209354	Translation initiation factor eIF-2B subunit alpha	3.1
83840	Ribosomal protein S6 kinase beta-1	2.6
11080	DnaJ homolog subfamily B member 4	2.5
42672	Regulator of G-protein signaling loco	2.3
29542	Hippocampal cholinergic neurostimulating peptide	2.3
178659	Heat shock protein Hsp-16.2	−2.2
44801	Stress-activated protein kinase JNK	−2.5
39071	Heat shock protein 67B3	−2.7
34780	Mitogen-activated protein kinase 14B	−3.0
37744	Protein lethal(2)essential for life	−3.8
39077	Heat shock protein 23	−3.9
360006	DnaJ homolog subfamily A member 2	−4.3
37985	Transient receptor potential cation channel protein painless	−4.5

This GO category is significantly overrepresented in heart tissues as compared to muscle tissues (adj *p* < 0.05) and contains 18 DE genes out of 32 total annotated genes. Only DE genes with a log_2_fold change greater or less than 2 are displayed in this table

In particular, this analysis identified two transient receptor potential (TRP) channel genes, pyrexia and painless, that belong to TRP A family (Table 2). Full analysis of our transcriptome identified 5 subtypes of TRP channels including three types of TRP A family genes (TRPA1, pyrexia, and painless), TRP M2 and TRP M3. TRP channels are a diverse family of channels permeable to Na^+^ and/or Ca^2+^ and involved in many varied types of sensory reception. Of the TRP channels identified in our transcriptome, research in other models establishes TRP M3 as an osmoreceptor, M2 is a possible oxidant stress sensor, and TRP A genes in temperature sensing [88]. TRP A pyrexia and painless may be responsible for sensing different temperature ranges [89–92]. The differential expression of these channels in our samples, the upregulation of pyrexia in heart tissues and the upregulation of painless in CNS tissue, suggests a possible mechanism by which these systems are able to efficiently sense and react to temperature changes.

## Conclusions

Our study detected tissue-specific patterns of increased expression of neuromodulators in the heart (octopamine), muscle (myomodulin), and nervous system (RPCH, AST, tachykinin, FLRFamide), as well as tissue-specific variation in expression sequences for octopamine and NO. We identified several neuronal factors contributing to the identity of our abdominal and cardiac systems. For the abdominal network, we observed significantly increased expression of acetylcholine receptors, GluK2, voltage gated L-type Ca^2+^ channels, calcium-activated Cl^−^ channels, and K^+^ channels slo-1/slowpoke, and significantly increased expression of neuromodulators CCAP and AST. For the cardiac network, we observed increased expression of the neuromodulators myomodulin and octopamine, and GluK3, Gluk5, and IRK channels. We identified a set of neuronal differentiation transcription factors in these systems, including ceh-36, ttx-1, ets-5 and Shox2. This research also elucidated a novel role for the neuromodulator proctolin in regulating immune responses, and identified an overrepresentation of response to heat in the heart tissues of the lobsters, and suggests a possible mechanism for thermoreception in this poikilotherm stemming from thermosensory TRP A channels in the nervous system tissues.

## Methods

### Sample collection and preparation

Adult *Homarus americanus* were purchased from local commercial fisherman (F/V Jacqueline Bess, Nahant Fish and Lobster, Nahant, MA) and held in ambient running seawater tanks (10.29 ± 0.01 °C) at the Northeastern University Marine Science Center. Lobsters were not fed prior to experimentation. Four different tissue types were collected from live *Homarus americanus*: (1) abdominal nerve cord (*n* = *3*), (2) supraesophogeal ganglion (the “brain”) (*n = 1*), (3) heart and pericardial cavity with neurosecretory pericardial organ (*n = 3*), (4) muscle tissue from abdominal muscles (*n = 3*). Prior to dissection, individuals were subjected to once-daily hormone treatments of the proctolin (Arg-Tyr-Leu-Pro-Thr) (injection of proctolin in physiological saline) or control treatments (physiological saline injection) for a three-day period. Hormone treatments served to temporarily increase systemic proctolin concentration to 10^−6^ M, based on standard hemolymph/bodyweight calculations [93] and physiologically relevant concentration levels [13, 45, 47]. Efficacy of injections increasing systemic proctolin was confirmed by quantitative mass spectrometry. The period of treatment was selected based on time frames of ion channel turnover, a process that can take hours to days [94–98]. Samples were removed with forceps and surgical scissors, flash-frozen in TRI® Reagent with liquid nitrogen, and pulverized with RNase-DNase-free pestles (VWR, Radnor, PA). We used Agilent Bioanalyzer 2100 to QC total RNA before library preparation, and visually inspected the RNA peaks and fluorescence units. To prep samples for RNA-Seq on the Illumina platform, mRNA was separated from the extracted Total RNA with Dynabeads® Oligo(dT)_25_ (Invitrogen Life Technologies, Grand Island, NY). cDNA libraries were constructed using the Vollmer laboratory protocol for non-genetic model organisms [99–101] and NEBNext® reagents for Illumina® (New England Biolabs, Ipswich, MA). Samples were barcoded, multiplexed, and sequenced (single-end, 109bp) in a single lane on the Illumina HiSeq2000 platform at Tufts TUCF Genomics (Boston, MA).

### Data processing

RNA Sequencing on the Illumina HiSeq2000 platform yielded 145.3 million total reads, averaging 14.5 million reads per sample. Reads were trimmed and quality controlled to a Phred score of Q = 30. Read contamination was resolved prior to assembly by aligning all raw reads, using Bowtie (version 0.12.7) and custom perl scripts, against several indices and retaining only unmapped reads. These indices were constructed from the following inputs: (1) metazoan rRNA databases (NCBI), (2) viral, fungal, and bacterial genomes (NCBI), and (3) Illumina TruSeq adaptor sequences and PCR primers. Next, *de novo* assembly of the transcriptome was conducted in *Trinity* (version 8-14-2013), allowing for the detection of punitive alternative splice variants [102, 103]. Subsequently, contigs from our transcriptome were BLAST annotated against NCBI, UniProtKB’s Swiss-Prot, and TrEMBLE protein databases; matches with an e-value below 10^−4^ were considered protein-coding genes. Reads were aligned using Bowtie and include all partial and multiple alignments. Bowtie alignments of our dataset against selected genes from major signaling pathways as well as housekeeping genes, revealed high efficiency in gene recovery compared to available genomes of closely related species. 110.7 million reads mapped to our transcriptome and were used in gene expression analysis. Of these mapped reads, 66 % mapped to annotated transcripts. Blast output was parsed to the top hit for each contig, and NCBI gi numbers were converted to Entrez Gene IDs using ID mapping files from UniProt Knowledgebase (UniProtKB). All gene ID numbers provided are Entrez Gene IDs unless otherwise specified. Moreover, Entrez Gene IDs were mapped to gene ontology (GO) annotations using NCBI gene2go.

Differences in gene expression for all transcripts were analyzed from raw read counts using the R package DESeq2 1.4.5 [104] and merged with annotation files (for breakdown of analyses, see Additional file 1: Table S1). Results from differential expression analysis were further analyzed for difference in expression of gene ontologies using the R package GOseq (version 1.16.2), which determines expression of gene ontology categories accounting for over-detection of differential expression for long or highly expressed transcripts [105]. As multiple contigs often mapped to the same protein annotation, enrichment analysis was conducted on contigs with greatest read coverage for each given Entrez Gene ID. GOseq was run using gene annotation length as bias data and our GO category mapping file. *P*-values for overrepresented GO categories were adjusted using the Benjamini and Hochberg (1995) method for false discovery rate control. GO enrichment was visualized in REVIGO using GOseq output [106].

### Ethics approval

*Homarus americanus* is not an endangered or protected species, and ethical approval was not required for these experiments. Experimental procedures were conducted according best practices in the *Homarus americanus* community with efforts to minimize discomfort or distress.

### Availability of supporting data

The reference transcriptome sequences are available on BioProject [accession number PRJNA297570]. Additionally, the *Homarus americanus* transcriptome, annotations, and raw read count data (doi:10.5061/dryad.h617h) are available on Dryad.

## Additional files

Additional file 1: Table S1. Experimental design for represented differential gene expression analyses conducted in DESeq2. **Table S2.** Terminal selector genes differentially expressed in heart tissue compared to muscle tissue and central nervous system tissue (supraesophogeal ganglion, abdominal ganglion) compared to muscle tissue. Count indicates the total number of transcripts annotating to proteins (e-val < 10^−4^) identified as transcription factors, co-factors, or other regulatory genes involved in differentiating the terminal identity of a neuron type. Columns denote the total number of DE transcripts (DE, *p* < 0.05), and number of upregulated (up) and downregulated (down) DE transcripts in contrasts: heart vs. muscle tissue and nervous system vs. muscle tissue. **Table S3.** Gene ontology analysis of abdominal nerve cord vs. heart tissue. Analysis includes only significantly enriched GO terms (adj *p* value < 0.05) and depicts the ‘count’ or number contigs mapping to genes in the GO category, number of DE contigs (adj *p* < 0.05), DE contigs upregulated (“up”, log_2_foldchange > 0 or downregulated (“down”, log_2_foldchange < 0) in the abdominal ganglia compared to the heart ganglia. **Table S4.** Differentially expressed membrane channels in the abdominal ganglia compared to the heart. Table includes only DE contigs with a log2fold changes greater than four or less than negative four. (DOCX 111 kb) Additional file 2: Figure S1. Heatmap display of sample-to-sample Euclidean distances between samples, calculated by regularized log transformation. (TIFF 1415 kb) Additional file 3: Figure S2. Treemap of enriched biological process GO categories in heart tissues compared to muscle tissues. Box size indicates relative level of overrepresentation in heart tissues. The only significantly overrepresented biological process was response to heat (GO: 0009408), with over 50 % of involved genes differentially expressed (18 of 32 genes in category, *p* < 0.05). (JPG 1509 kb)

## Abbreviations

CPG: Central pattern generator
DE: Differentially expressed
GO: Gene ontology
SO: Significantly overrepresented
NO: Neuronal nitric oxide synthase
TRP channel: Transient receptor potential channel
GPCR: G-protein coupled receptor
IRK channel: Inward rectifying potassium channel
