# Supplementary material for: De novo transcriptome assembly for the lobster Homarus americanus and characterization of differential gene expression across nervous system tissues
Source: BMC Genomics. 2016 Jan 16;17:63. doi: 10.1186/s12864-016-2373-3 (PMC4715275; doi:10.1186/s12864-016-2373-3)
Supplement: Additional file 1: Table S1. — Experimental design for represented differential gene expression analyses conducted in DESeq2. Table S2. Terminal selector genes differentially expressed in heart tissue compared to muscle tissue and central nervous system tissue (supraesophogeal ganglion, abdominal ganglion) compared to muscle tissue. Count indicates the total number of transcripts annotating to proteins (e-val < 10−4) identified as transcription factors, co-factors, or other regulatory genes involved in differentiating the terminal identity of a neuron type. Columns denote the total number of DE transcripts (DE, p < 0.05), and number of upregulated (up) and downregulated (down) DE transcripts in contrasts: heart vs. muscle tissue and nervous system vs. muscle tissue. Table S3. Gene ontology analysis of abdominal nerve cord vs. heart tissue. Analysis includes only significantly enriched GO terms (adj p value < 0.05) and depicts the ‘count’ or number contigs mapping to genes in the GO category, number of DE contigs (adj p < 0.05), DE contigs upregulated (“up”, log2foldchange > 0 or downregulated (“down”, log2foldchange < 0) in the abdominal ganglia compared to the heart ganglia. Table S4. Differentially expressed membrane channels in the abdominal ganglia compared to the heart. Table includes only DE contigs with a log2fold changes greater than four or less than negative four. (DOCX 111 kb) [file 12864_2016_2373_MOESM1_ESM.docx]

**Supplementary Tables**

**Table S1.** Experimental design for represented differential gene expression analyses conducted in DESeq2.

| **Analysis** | **Design** | **Contrast(s)** |
| --- | --- | --- |
| Treatment | ~ Treatment | Treated vs. Untreated |
| Tissue Type – Combined Nerve Tissues *(Abdominal + Supraesophogeal Ganglia)* | ~ Treatment + Tissue Type | Nerve vs. Muscle  Heart vs. Muscle  Nerve vs. Heart |
| Tissue Type – Separate Nerve Tissues  *(Supraesophogeal Ganglia removed)* | ~ Treatment + Tissue Type | Abdominal Ganglia vs. Heart |

**Table S2.** Terminal selector genes differentially expressed in heart tissue compared to muscle tissue and central nervous system tissue (supraesophogeal ganglion, abdominal ganglion) compared to muscle tissue. Count indicates the total number of transcripts annotating to proteins (e-val < 10^-4^) identified as transcription factors, co-factors, or other regulatory genes involved in differentiating the terminal identity of a neuron type. Columns denote the total number of DE transcripts (DE, *p* < 0.05), and number of upregulated (up) and downregulated (down) DE transcripts in contrasts: heart vs. muscle tissue and nervous system vs. muscle tissue.

|  | |  | ***Heart*** | | | ***Nervous System*** | | |  |  |  |
| --- | --- | --- | --- | --- | --- | --- | --- | --- | --- | --- | --- |
| ***Gene*** | ***count*** | | ***DE*** | ***Up*** | ***Down*** | ***DE*** | ***Up*** | ***Down*** | ***Cell Fate*** | ***Type*** | ***Reference*** |
| *ceh-36* | | 3 | 1 | 1 | 0 | 2 | 2 | 0 | AWC sensory neuron | Otx-type homeodomain | Serrano-Saiz et al. 2013 |
| *ttx-1* | | 9 | 4 | 3 | 1 | 5 | 4 | 1 | AFD sensory neuron | Otx-type/LIM homeodomain | Serrano-Saiz et al. 2013 |
| *Lhx7/Lhx8* | | 30 | 14 | 2 | 12 | 16 | 2 | 14 | GABAergic or cholinergic neurons | LIM homeodomain protein | Fragkouli et al. 2009 |
| *Isl1* | | 48 | 20 | 10 | 10 | 22 | 9 | 13 | GABAergic or cholinergic neurons | LIM homeodomain protein | Fragkouli et al. 2009 |
| *lin-11* | | 62 | 25 | 9 | 16 | 27 | 15 | 12 | ASG sensory neuron, ADL sensory neuron | LIM homeodomain ceh-37 Otx-type | Serrano-Saiz et al. 2013 |
| *Etv1/erb81* | | 28 | 9 | 9 | 0 | 12 | 12 | 0 | Dopaminergic neurons | ETS transcription factor | Flames and Hobert 2009 |
| *pet-1/FEV* | | 24 | 8 | 8 | 0 | 10 | 10 | 0 | Serotonergic neurons | Cascade factor | Liu et al. 2010 |
| *ets-5* | | 24 | 8 | 8 | 0 | 10 | 10 | 0 | BAG sensory neuron | Ets + ceh-37 Otx-type homeodomain | Serrano-Saiz et al. 2013 |
| *AST-1* | | 24 | 8 | 8 | 0 | 10 | 10 | 0 | All dopaminergic neurons | ETS-type transcription factor | Hobert 2008 |
| *Lhx6* | | 56 | 26 | 8 | 18 | 22 | 8 | 14 | GABAergic or cholinergic neurons, sensory neurons | LIM homeodomain protein | Fragkouli et al. 2009 |
| *unc-42* | | 34 | 10 | 9 | 1 | 13 | 11 | 2 | PLM sensory neuron | Prd-type homeodomain | Serrano-Saiz et al. 2013 |
| *TTX-3/CEH-10* | | 21 | 5 | 4 | 1 | 8 | 6 | 2 | AIY interneurons | LIM/Prd homeodomain dimer | Hobert 2008 |
| *UNC-30* | | 22 | 6 | 5 | 1 | 8 | 6 | 2 | GABAergic ventral cord motorneurons | Prd-type homeodomain | Hobert 2008 |
| *vab-3* | | 23 | 5 | 4 | 1 | 8 | 6 | 2 | OLL and URY sensory neurons | Prd homeodomain, Pax homeodomain | Serrano-Saiz et al. 2013 |
| *ceh-14* | | 62 | 20 | 9 | 11 | 21 | 16 | 5 | PHC, PHB, PHA sensory neurons, DVC interneuron | LIM homeodomain | Serrano-Saiz et al. 2013 |
| *mec-3* | | 27 | 8 | 8 | 0 | 9 | 9 | 0 | Mechanosensory neurons, FLP and PVD sensory neurons | LIM homeodomain | Serrano-Saiz et al. 2013, Hobert 2008 |
| *ceh-43* | | 20 | 5 | 4 | 1 | 6 | 5 | 1 | Dopaminergic neurons | Factor | Doitsidou et al. 2013 |
| *ttx-3* | | 33 | 10 | 9 | 1 | 8 | 7 | 1 | ASK sensory neuron | LIM homeodomain | Serrano-Saiz et al. 2013 |
| *Gata2* | | 13 | 5 | 5 | 0 | 3 | 3 | 0 | GABAergic neurons | Postmitotic selector gene | Virolainen et al. 2012 |
| *ceh-20* | | 21 | 3 | 0 | 3 | 2 | 0 | 2 | Dopaminergic neurons | Pbx factor | Doitsidou et al. 2013 |
| *che-1* | | 501 | 23 | 13 | 10 | 44 | 23 | 21 | ASE sensory neuron | Zinc finger transcription factor | Serrano-Saiz et al. 2013 |
| *ceh-40* | | 16 | 2 | 0 | 2 | 1 | 0 | 1 | Dopaminergic neurons | Pbx factor | Doitsidou et al. 2013 |
| *unc-86* | | 5 | 0 | 0 | 0 | 0 | 0 | 0 | AIZ, AIM interneurons | POU/LIM homeodomain | Serrano-Saiz et al. 2013 |
| *ceh-6* | | 5 | 0 | 0 | 0 | 0 | 0 | 0 | AUA interneuron | POU homeodomain | Serrano-Saiz et al. 2013 |

**Table S3.** Gene ontology analysis of abdominal nerve cord vs. heart tissue. Analysis includes only significantly enriched GO terms (adj *p* value < 0.05) and depicts the ‘count’ or number contigs mapping to genes in the GO category, number of DE contigs (adj *p* < 0.05), DE contigs upregulated (“up”, log_2_foldchange > 0 or downregulated (“down”, log_2_foldchange < 0) in the abdominal ganglia compared to the heart ganglia.

| ***GO ID*** | ***GO Term*** | ***Category*** | ***Count*** | ***DE*** | ***Up*** | ***Down*** |
| --- | --- | --- | --- | --- | --- | --- |
| GO:0005886 | plasma membrane | Component | 3069 | 883 | 781 | 102 |
| GO:0016021 | integral component of membrane | Component | 3759 | 751 | 599 | 152 |
| GO:0070062 | extracellular vesicular exosome | Component | 1806 | 377 | 293 | 84 |
| GO:0005576 | extracellular region | Component | 679 | 217 | 177 | 40 |
| GO:0005615 | extracellular space | Component | 570 | 159 | 132 | 27 |
| GO:0031012 | extracellular matrix | Component | 240 | 117 | 110 | 7 |
| GO:0005578 | proteinaceous extracellular matrix | Component | 221 | 84 | 75 | 9 |
| GO:0031225 | anchored component of membrane | Component | 102 | 52 | 50 | 2 |
| GO:0005604 | basement membrane | Component | 140 | 56 | 44 | 12 |
| GO:0042383 | sarcolemma | Component | 108 | 54 | 35 | 19 |
| GO:0043195 | terminal bouton | Component | 72 | 35 | 31 | 4 |
| GO:0016529 | sarcoplasmic reticulum | Component | 57 | 21 | 9 | 12 |
| GO:0005747 | mitochondrial respiratory chain complex I | Component | 30 | 27 | 0 | 27 |
| GO:0005840 | ribosome | Component | 114 | 42 | 0 | 42 |
| GO:0070469 | respiratory chain | Component | 21 | 18 | 0 | 18 |
| GO:0005763 | mitochondrial small ribosomal subunit | Component | 29 | 23 | 0 | 23 |
| GO:0007155 | cell adhesion | Process | 618 | 203 | 178 | 25 |
| GO:0007411 | axon guidance | Process | 552 | 175 | 157 | 18 |
| GO:0055085 | transmembrane transport | Process | 669 | 160 | 146 | 14 |
| GO:0030198 | extracellular matrix organization | Process | 246 | 76 | 72 | 4 |
| GO:0005975 | carbohydrate metabolic process | Process | 318 | 83 | 63 | 20 |
| GO:0007274 | neuromuscular synaptic transmission | Process | 77 | 36 | 34 | 2 |
| GO:0030574 | collagen catabolic process | Process | 47 | 27 | 26 | 1 |
| GO:0006412 | translation | Process | 252 | 97 | 2 | 95 |
| GO:0005509 | calcium ion binding | Function | 870 | 246 | 219 | 27 |
| GO:0003779 | actin binding | Function | 585 | 184 | 131 | 53 |
| GO:0003824 | catalytic activity | Function | 459 | 105 | 66 | 39 |
| GO:0008307 | structural constituent of muscle | Function | 134 | 74 | 17 | 57 |
| GO:0004129 | cytochrome-c oxidase activity | Function | 24 | 16 | 1 | 15 |
| GO:0003735 | structural constituent of ribosome | Function | 133 | 80 | 0 | 80 |
| GO:0008137 | NADH dehydrogenase (ubiquinone) activity | Function | 19 | 18 | 0 | 18 |

**Table S4.** Differentially expressed membrane channels in the abdominal system compared to the heart (adj *p* < 0.05). Table includes only DE contigs with a log_2_fold changes greater than four or less than negative four.

| ***GeneID*** | ***Annotation*** | ***Abbreviation*** | ***Type*** | ***Log_2_Fold***  ***Change*** |
| --- | --- | --- | --- | --- |
| 22802 | Calcium-activated chloride channel regulator 4 | CLCA4 | Cl | 7.2 |
| 781 | Voltage-dependent calcium channel subunit alpha-2/delta-1 | CA2D1 | Ca | 6.9 |
| 32020 | Open rectifier potassium channel protein 1 | ORK1 | K | 6.7 |
| 42350 | Glutamate-gated chloride channel | GLUCL | Cl | 6.3 |
| 783 | Voltage-dependent L-type calcium channel subunit beta-2 | CACB2 | Ca | 6.2 |
| 42940 | Calcium-activated potassium channel slowpoke | SLO | K | 5.9 |
| 783 | Voltage-dependent L-type calcium channel subunit beta-2 | CACB2 | Ca | 5.9 |
| 23844 | Calcium-activated chloride channel regulator 1 mCLCA3 | CLCA1 | Cl | 5.8 |
| 9635 | Calcium-activated chloride channel regulator 2 | CLCA2 | Cl | 5.8 |
| 784768 | Epithelial chloride channel protein | ECLC | Cl | 5.7 |
| 37985 | Transient receptor potential cation channel protein painless | PAIN | Cation | 5.7 |
| 65272 | Potassium channel subfamily K member 10 | KCNKA | K | 5.7 |
| 574295 | Calcium-activated chloride channel regulator 1 | CLCA1 | Cl | 5.6 |
| 32020 | Open rectifier potassium channel protein 1 | ORK1 | K | 5.4 |
| 41428 | Chloride channel protein 2 ClC-2 | CLCN2 | Cl | 5.4 |
| 781 | Voltage-dependent calcium channel subunit alpha-2/delta-1 | CA2D1 | Ca | 5.4 |
| 785 | Voltage-dependent L-type calcium channel subunit beta-4 | CACB4 | Ca | 5.3 |
| 180203 | Calcium-activated potassium channel slo-1 | SLO1 | K | 5.3 |
| 32619 | Sodium channel protein para | SCNA | Na | 5.2 |
| 22802 | Calcium-activated chloride channel regulator 4 | CLCA4 | Cl | 5.1 |
| 42350 | Glutamate-gated chloride channel | GLUCL | Cl | 5.1 |
| 785 | Voltage-dependent L-type calcium channel subunit beta-4 | CACB4 | Ca | 5.1 |
| 22802 | Calcium-activated chloride channel regulator 4 | CLCA4 | Cl | 5.1 |
| NA | Aquaporin | AQP | Water | 5.0 |
| 408764 | Voltage-dependent calcium channel type A subunit alpha-1 | CAC1A | Ca | 4.9 |
| 9635 | Calcium-activated chloride channel regulator 2 | CLCA2 | Cl | 4.8 |
| 42350 | Glutamate-gated chloride channel | GLUCL | Cl | 4.7 |
| 32619 | Sodium channel protein para | SCNA | Na | 4.6 |
| 229933 | Calcium-activated chloride channel regulator 2 | CLCA2 | Cl | 4.6 |
| 42940 | Calcium-activated potassium channel slowpoke | SLO | K | 4.6 |
| 32619 | Sodium channel protein para | SCNA | Na | 4.5 |
| NA | Voltage-gated potassium channel subunit beta-2 | KCAB2 | K | 4.5 |
| 41428 | Chloride channel protein 2 ClC-2 | CLCN2 | Cl | 4.4 |
| 445371 | Potassium channel subfamily K member 18 | KCNKI | K | 4.4 |
| 242735 | BK channel auxilliary gamma subunit LRRC38 | LRC38 | K | 4.3 |
| 23844 | Calcium-activated chloride channel regulator 1 mCLCA3 | CLCA1 | Cl | 4.2 |
| 9635 | Calcium-activated chloride channel regulator 2 | CLCA2 | Cl | 4.1 |
| 42940 | Calcium-activated potassium channel slowpoke | SLO | K | 4.1 |
| 229933 | Calcium-activated chloride channel regulator 2 | CLCA2 | Cl | 4.0 |
| NA | Voltage-gated potassium channel subunit beta-3 | KCAB3 | K | 4.0 |
| 9635 | Calcium-activated chloride channel regulator 2 | CLCA2 | Cl | 4.0 |
| 40129 | Potassium voltage-gated channel protein Shal | KCNAL | K | 3.9 |
| 42940 | Calcium-activated potassium channel slowpoke | SLO | K | 3.9 |
| NA | Chloride channel protein 2 | CLCN2 | Cl | 3.8 |
| 32619 | Sodium channel protein para | SCNA | Na | 3.8 |
| NA | Aquaporin | AQP | Water | 3.8 |
| 181139 | TWiK family of potassium channels protein 18 | TWK18 | K | 3.8 |
| 32619 | Sodium channel protein para | SCNA | Na | 3.8 |
| 180203 | Calcium-activated potassium channel slo-1 | SLO1 | K | 3.8 |
| 784768 | Epithelial chloride channel protein | ECLC | Cl | 3.8 |
| 93589 | Voltage-dependent calcium channel subunit alpha-2/delta-4 | CA2D4 | Ca | 3.7 |
| 41428 | Chloride channel protein 2 ClC-2 | CLCN2 | Cl | 3.7 |
| 32619 | Sodium channel protein para | SCNA | Na | 3.7 |
| 781 | Voltage-dependent calcium channel subunit alpha-2/delta-1 | CA2D1 | Ca | 3.7 |
| 42350 | Glutamate-gated chloride channel | GLUCL | Cl | 3.6 |
| 12294 | Voltage-dependent calcium channel subunit alpha-2/delta-3 | CA2D3 | Ca | 3.6 |
| 408764 | Voltage-dependent calcium channel type A subunit alpha-1 | CAC1A | Ca | 3.6 |
| 229933 | Calcium-activated chloride channel regulator 2 | CLCA2 | Cl | 3.6 |
| 100008648 | ATP-sensitive inward rectifier potassium channel 11 | IRK11 | K | -3.5 |
| 494803 | Potassium channel subfamily K member 9 | KCNK9 | K | -3.9 |
| 294141 | Chloride intracellular channel protein 2 | CLIC2 | Cl | -3.9 |
| 16515 | ATP-sensitive inward rectifier potassium channel 12 | IRK12 | K | -4.0 |
| 9635 | Calcium-activated chloride channel regulator 2 | CLCA2 | Cl | -4.0 |
| 23844 | Calcium-activated chloride channel regulator 1 mCLCA3 | CLCA1 | Cl | -4.2 |
| 29712 | Inward rectifier potassium channel 2 | IRK2 | K | -4.3 |
| 100008648 | ATP-sensitive inward rectifier potassium channel 11 | IRK11 | K | -4.5 |
| 817099 | Potassium channel AKT6 | AKT6 | K | -4.8 |
| NA | PREDICTED: epithelial chloride channel protein-like | predicted | Cl | -6.4 |
