# Supplementary figures and images for: De novo transcriptome assembly for the lobster Homarus americanus and characterization of differential gene expression across nervous system tissues
Source: BMC Genomics. 2016 Jan 16;17:63. doi: 10.1186/s12864-016-2373-3 (PMC4715275; doi:10.1186/s12864-016-2373-3)

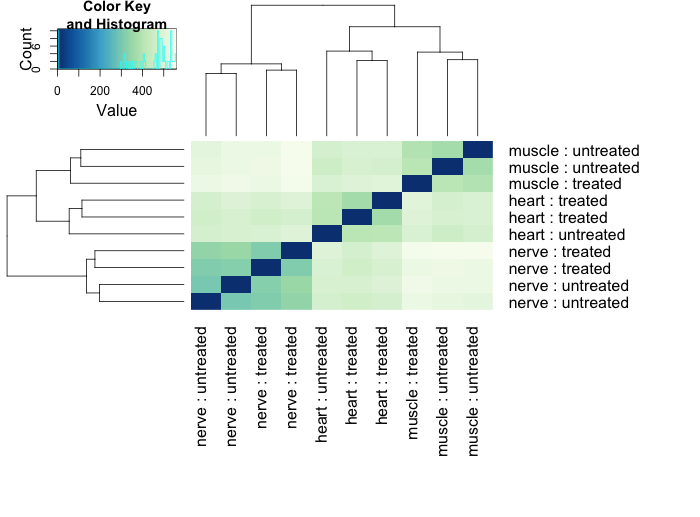

Supplement: Additional file 2: Figure S1. — Heatmap display of sample-to-sample Euclidean distances between samples, calculated by regularized log transformation. (TIFF 1415 kb) [file 12864_2016_2373_MOESM2_ESM.tiff]

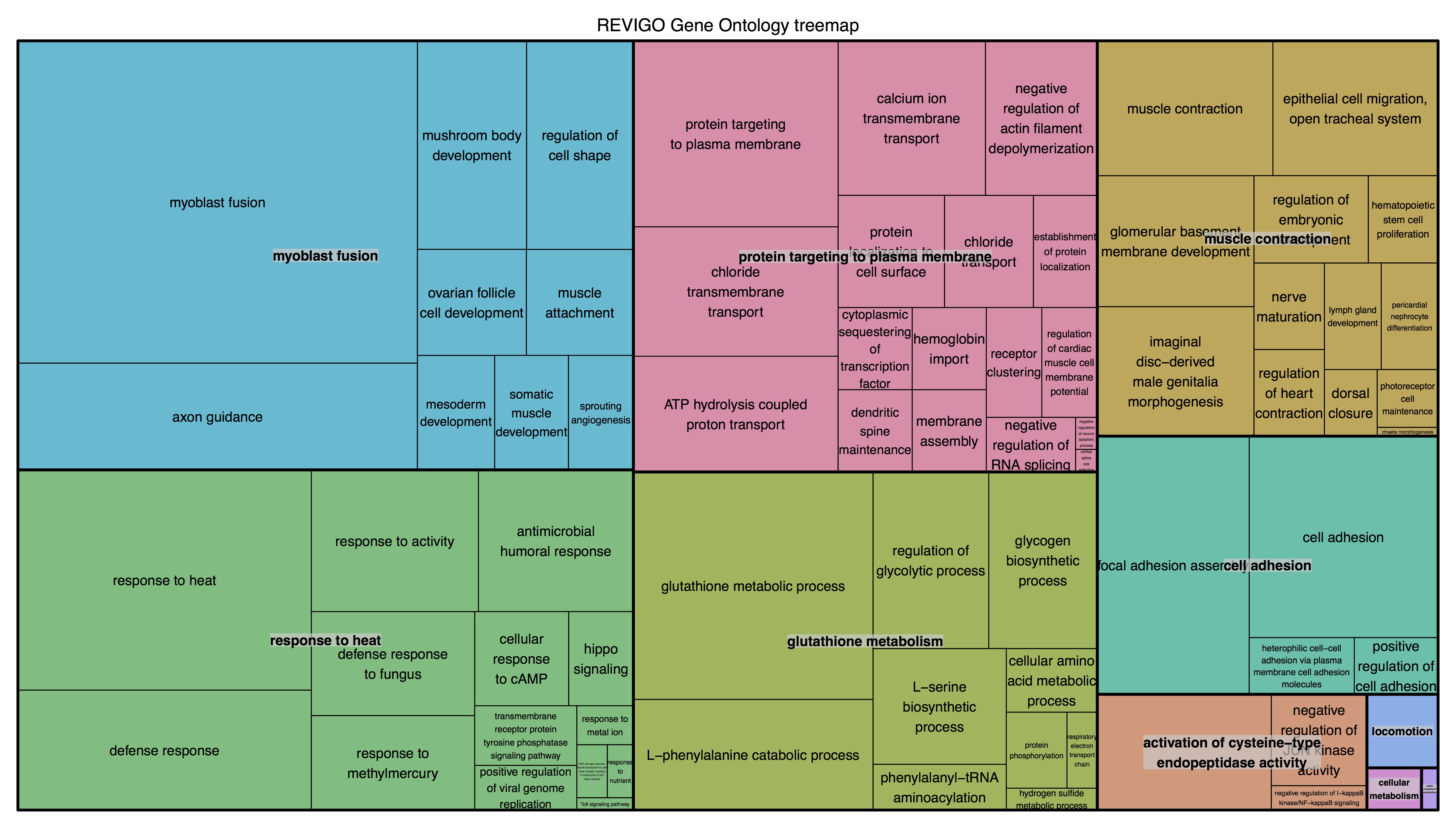

Supplement: Additional file 3: Figure S2. — Treemap of enriched biological process GO categories in heart tissues compared to muscle tissues. Box size indicates relative level of overrepresentation in heart tissues. The only significantly overrepresented biological process was response to heat (GO: 0009408), with over 50 % of involved genes differentially expressed (18 of 32 genes in category, p < 0.05). (JPG 1509 kb) [file 12864_2016_2373_MOESM3_ESM.jpg]
